# A Realist Evaluation of Case Management Models for People with Complex Health Conditions Using Novel Methods and Tools—What Works, for Whom, and under What Circumstances?

**DOI:** 10.3390/ijerph20054362

**Published:** 2023-02-28

**Authors:** Sue Lukersmith, Luis Salvador-Carulla, Younjin Chung, Wei Du, Anoush Sarkissian, Michael Millington

**Affiliations:** 1 Health Research Institute, University of Canberra, Canberra 2617, Australia; 2 Lukersmith & Associates, Sydney 2777, Australia; 3 Centre for Mental Health Research, Australian National University, Canberra 2601, Australia; 4 School of Public Health, Southeast University, Nanjing 211189, China; 5 Wellbeing Rehab, Sydney 2112, Australia; 6 Centre for Disability Studies, University of Sydney, Sydney 2006, Australia

**Keywords:** case manager, case management taxonomy, severe injury, realist evaluation, person-centred case management, generalist case management, pattern analysis

## Abstract

Case management developed from a generalist model to a person-centred model aligned with the evidence-informed evolution of best practice people-centred integrated care. Case management is a multidimensional and collaborative integrated care strategy where the case manager performs a set of interventions/actions to support the person with a complex health condition to progress in their recovery pathway and participate in life roles. It is currently unknown what case management model works in real life for whom and under what circumstances. The purpose of this study was to answer these questions. The study methods used realistic evaluation framework, examined the patterns and associations between case manager actions (mechanisms), the person’s characteristics and environment (context), and recovery (outcomes) over 10 years post severe injury. There was mixed methods secondary analysis of data extracted via in-depth retrospective file reviews (*n* = 107). We used international frameworks and a novel approach with multi-layered analysis including machine learning and expert guidance for pattern identification. The study results confirm that when provided, a person-centred case management model contributes to and enhances the person’s recovery and progress towards participation in life roles and maintaining well-being after severe injury.Furthermore, the intensity of case management for people with traumatic brain injury, and the person-centred actions of advising, emotional and motivational support, and proactive coordination contribute to the person achieving their goals. The results provide learnings for case management services on the case management models, for quality appraisal, service planning, and informs further research on case management.

## 1. Introduction

The evidence-informed evolution of person- and people-centred integrated care has developed over the last decade as an international best practice standard [1,2,3,4]. The World Health Organization Global Strategy on Integrated people-centred health services identified community-based case management (CM) as a key pillar for integrated care [4,5]. There are many names for, and definitions of case management [6]. The World Health Organization and The King’s Fund define case management as a targeted, community-based and proactive approach to care that involves case-finding, assessment, care planning, and care coordination to integrate services around the needs of people with long-term conditions [4,7]. This study used a framework to classify the actions of community-based case managers—the Case Management Taxonomy (CMTaxonomy) [8]. The CMTaxonomy defines case management based in the community as a multidimensional and collaborative process. It involves a set of interventions and actions (mechanisms) for assessment, planning, coordinating, and review of the options and services required to meet the client’s health-related needs, and support them to reach their goals related to participation in life roles [6,9].

CM has evolved into different models. One model is the *Generalist/Broker* (Generalist) model. Aligned with the international trend towards integrated and person-centred care another is the *Person-centred community-based model* (Person-centred) model. The Generalist model is described as focused on the practical or operational needs of the person and is an administrative andstructured approach. The focus is on actions to meet the person’s needs through brokered services. The Person-centred model includes all actions from the Generalist CM actions. However, this model also uses a proactive and preventive approach with a focus on the person’s needs and motivations, involves planning of participation goals, and utilises the person’s strengths. The Person-centred model also involves managing some contextual factors which become barriers to—or facilitators toward—achieving goals (e.g., stable accommodation).

Evaluation of the various models of CM for people with complex health conditions, such as brain injury, is lacking (including for people with traumatic brain injury [6,10,11,12,13]. The evidence suggests that case management for people with brain injury is needed even though it represents a high cost. The involvement of a case manager can enhance the collaboration, integration, and coordination of cross sector care (health, social, education) to meet the needs of the person and their family [12,14,15,16]. However, evidence of each model’s effectiveness and consideration of the different CM actions performed is limited for people with complex conditions. The lack of evidence is a barrier on progress towards the implementation and quality appraisal of the most effective best practice Person-centred CM model. The problems are: (1) Finding what works, for whom and under what circumstances; and (2) Utilising tools fit for purpose to measure and analyse CM actions.

### 1.1. Study Setting

In 2018, a public social insurance organisation in New South Wales (NSW) Australia, the Lifetime Care and Support Scheme (the Scheme, which is part of icare NSW), sought to undertake a realist evaluation of CM. The Scheme funds the treatment, rehabilitation, and support (e.g., personal care) for people who have complex health conditions as a consequence of severe injuries—specifically burns, amputations, traumatic brain injury (TBI), or spinal cord injury (SCI)—sustained in a motor vehicle crash. External case managers (approved health professionals with the necessary experience and who meet service quality standards) are contracted to provide CM to the injured person (hereafter referred to as participant). CM in the Scheme is a high cost typically around 6% annually of the Scheme’s total costs [17].

### 1.2. Research Aim and Objectives

The aim of this study was to retrospectively describe and examine the case management provided to Scheme participants. The objectives were to:

1. Describe the circumstances of the participant (context), the CM actions (mechanism), the CM model and the participant outcomes (outcomes).
2. Examine patterns and associations between these components and the participant’s recovery pathway across the 10 years following injury.
3. Examine which of the different CM actions (and in turn CM model) are associated with participant outcomes.

## 2. Methods

### 2.1. Research Design

The study involved a mixed-methods retrospective secondary analysis of participant data extracted from the Scheme files. We utilised validated research methods including a realist evaluation (research design), three international frameworks (conceptual and coding models), and a novel multi-layered analysis (data analysis methods). Realist evaluations and associated program theory use the Context, Mechanism, Outcome configuration (C-M-O) a valuable approach to examine complex interventions [18,19]. This allows researchers to explore “what works for whom and under what circumstances to facilitate understanding of how different outcomes for people in different circumstances occur [18]. Our multi-layered data analysis methods used mapping and coding of complex variable information in the C-M-O configuration which could then be analysed for patterns and associations.

### 2.2. Time Frames and Ethical Approval

Data collection through in-depth file reviews occurred between July 2018–March 2019, the data analysis thereafter in different periods across 2019–2020. The internal report for the funder was written in 2021 [20]. The journal manuscript was prepared in 2022 when key authors had relocated to the University of Canberra.

Participants of the Lifetime Care and Support Scheme sign a consent form at the time of application into the Scheme. The consent includes the use of their personal and health information, and specifically states that their personal and health information may be used for the ‘research or the compilation of statistics’ including ‘program evaluation and research, service development activities’ such as this study. File reviews commenced in 2018 under the consent provided to the Scheme, with data extraction performed by SL and AS (who were existing sub-contractors to the Scheme). In 2019, the Australian National University (ANU) became involved in the study for the descriptive statistical and SOMNet (machine learning) analysis. Ethical approval was sought and approved in March 2019 (ANU Human Ethics Research Committee, #2019/15). The personal data extracted from participant files and provided to ANU were de-identified.

### 2.3. Participants and Procedures

**Figure 1:**
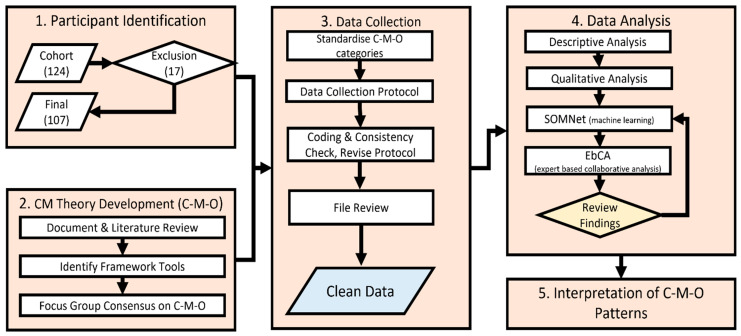
Flow chart of study method.

Figure 1 provides an overview of the methods for this study with further details described below.

#### 2.3.1. Participant Identification

There were (*n* = 124) people severely injured in motor vehicle crashes between 1 October 2007 and 30 September 2008 and who were accepted into the Lifetime Care and Support Scheme. Inclusion criteria for the study were: participants who had continued involvement in the Scheme for 5 years or more (thus there was recent information on their file); were adults at the time of the study; had sustained either a TBI or SCI or both (*n* = 107).

#### 2.3.2. CM Theory Development: Realist Context–Mechanism–Outcome (C-M-O)

Numerous sources of information and existing tools, international frameworks, the tacit and experiential knowledge of case management experts were used to develop the CM program theory [21,22]. The steps to develop the theory are outlined below.

- *Document and literature review*: In preparation for the CM theory development, a review of relevant documents and literature was completed, and key elements identified. The documents included:

- Previous participant Scheme wide outcome reports [23,24].
- Trauma research review on outcomes [25,26].

The key elements were provided to the case management experts (refer to focus group step *c. Focus group* below). This information was also used to develop focus questions for the expert case managers workshop participants.

b. *Identify framework tools*: There was a large amount of information on the file of each participant from the past 10 years. The following frameworks were identified as tools to code relevant information extracted from the file reviews relevant to the theory of C-M-O:

- Appropriate data collection time points and the phase of the participant’s recovery were guided by the ‘My Plan’ framework of the Scheme’s conceptual model for the pathway following severe injury [27].
- A framework to categorize CM actions (mechanisms) and intensity was needed. The file reviewers mapped case managers’ actions and intensity outlined in reports and file notes, to a CM model at the time of data extraction. In 2016, the Scheme had already published case manager expectations [28,29] framed in terms of the CMTaxonomy [8,9]. In Table 1, the actions performed in the Generalist and Person-centred models of CM are mapped using the validated CMTaxonomy [8,9,30,31,32,33]. Refer to the CMTaxonomy toolkit: the Intervention tree and actions (Mechanisms) in Supplementary File S1, the Service tree (a domain of Context) in Supplementary File S2 and the Glossary in Supplementary File S3.

c. *Focus group consensus on the C-M-O*: Four subject matter experts and a facilitator developed the program theory for person-centred approaches in CM and specifically in the context of people severely injured. The subject matter experts from LTCS included the Senior Service Development Manager—CM, a regional manager (who also had lived experience), Service Development Officer—CM and a Team Leader of the coordinators (staff who contracted and monitored the CM provided to participants). The first author completed the workshop preparation and facilitated the workshops using the frameworks to focus questions to stimulate and scaffold discussion.

There were four consensus workshops with CM experts over six months. The frameworks were endorsed by the expert CM consensus group. Key concepts were framed in a biopsychosocial health perspective using the World Health Organization International Classification of Functioning Disability and Health (ICF) and framework (domains and definitions) [34]. The My Plan recovery pathway was also used to broadly structure the C-M-O over the 10 years following injury [35].

From these, the list of contextual factors, mechanisms (including CM actions) and outcomes were identified. The list was revised to include the characteristics where data could reasonably be retrospectively extracted from the participant’s file, by expert case manager file reviewers (Refer to Supplementary File S4).

#### 2.3.3. Data Collection

##### Standardise C-M-O Categories

The following frameworks were used to map, code, and thereby standardise the information variables relevant to C-M-O (Refer to Supplementary File S5 for more detail):

i. The two level classification code from the World Health Organization International Functioning Disability and Health (ICF) classification available on the ICF browser [36].
ii. Established ICF linking rules to map information to the ICF appropriately. Developed in 2016, the refinements to the ICF Linking Rules strengthen the potential for comparability of health information [37]. The file reviewers used the linking rules to map information to the ICF (e.g., target of the goals).
iii. The World Health Organization International Classification of Health Interventions [38].
iv. The CMTaxonomy action/intervention and service trees [8,9].
v. The My Plan phases of recovery post injury were incorporated into the C-M-O framework related to time periods [27,39]. The phases post injury refers to the typical pathway for people following a severe traumatic injury, such as brain injury and spinal cord injury. In the My Plan recovery pathway, the first phase (from the time of injury to approximately 2 years post injury) is broadly described as the ‘continued recovery phase’. In the continuing recovery phase, the participant continues to adjust to their injury and changed circumstances. There may be continued treatment and rehabilitation for the participant to settle into living at home (often following extensive acute inpatient hospital care and rehabilitation), and doing activities (often using restorative, adaptive, or learning different approaches to perform activities). In the next phase—occurring approximately in the 3–5 years post injury period—the My Plan pathway refers to this as the Participation phase of resuming participation in life roles. The participant is involved in their chosen activities and major life areas such as education, work, purposeful occupations, domestic responsibilities, community, and social life. The final phase is approximately 8–10 years after their injury. In this phase, the participant has moved on and living their life. The participant has reached a stage and rhythm of maintaining their health and wellbeing [27]. Hereafter, these phases are referred to as ***Recovery*** (continued adjustment, treatment/rehabilitation)**, *Participation*** (in life roles), and ***Maintainin****g* (life, health and wellbeing) phases respectively. The phase along the My Plan recovery pathway was an outcome variable (Recovery, Participation, Maintaining).
vi. The My Plan goal rating using a three step achievement (not achieved, partially achieved, achieved) [27].

##### Data Collection Protocol

The data were collected through deep retrospective participant file reviews. The participant’s file contains case manager and treating practitioner reports, file notes by Scheme staff and CM invoices which provide the time spent by the CM over a specified time. The file reviews were conducted by two experienced CM (first and fifth authors), both of whom are registered Occupational Therapists. A protocol and Excel spreadsheet were prepared for the in-depth file reviews. The protocol included all variables and categories, definitions, and resources (e.g., access to the ICF online search portal). Where relevant and possible, the variables reliant on mapping and coding of textual information into categories were embedded in the Excel spreadsheet (drop down choices in cells for some variables). The full description of each category was detailed in the metadata (refer to Supplementary Files S4 and S5). The two file reviewers completed training on the functions for the Scheme’s central database system for participants (Navigator^®^).

Data collection involved 647 variables from each participant file. Demographic, social, and injury related data were identified, and categories established where appropriate. Data on C-M-O were collected from the file at three time periods (2 years post injury, 3–5 years post injury, and 8–10 years post injury). Variables on service costs, and information on the disputes and claims lodged by the participant/their family in the three time periods were also collected. The variables included are listed in Supplementary File S4. There were context variables (*n* = 373), mechanism variables (total *n* = 156, of which *n* = 70 related to CM actions and *n* = 86 to costs), and outcome variables (*n* = 118). Some factors are considered both context and mechanism e.g., marriage or in a relationship reflects the participant’s living situation (context) and suggests the participant is likely to receive informal support (mechanism) from their partner. The analysis considered these cross-cutting context/mechanism variables.

The file review, service costs, disputes, and complaints data were aggregated into one analytic dataset in Excel with a unique personal identifier to track longitudinal changes for each study participant.

##### Coding and Consistency Check, Protocol Revised

There were three steps and protocol iterations to revise the data collection protocol and data entry rules. The first step was to develop consistency (files and data extraction completed together (*n* = 2). The second step involved independent file reviews, comparisons, and discussion (*n* = 10). The third step involved file reviews completed independently, where the first author checked for consistency (*n* = 5).

##### File Review

Data extraction was completed for each of the remaining participant’s electronic file at three time periods aligned to the approximate estimates of the phases in the My Plan recovery pathway: 2 years post injury, 3–5 years post injury (depending on date of injury), and 8–10 years post injury (depending on the date of the injury).

##### Clean Data

The data extracted on the Excel spreadsheet were cleaned and incomplete data cross checked with the participant’s electronic file.

#### 2.3.4. Data Analysis

The analysis involved multiple layers and novel analytic methods, due to the complexity and nature of the C-M-O data (predominantly categorisation rather than numeric). The standard descriptive statistical analysis was inadequate. First there was a descriptive analysis of the participant data, which informed subsequent analytical approaches. Second was a qualitative analysis. The descriptive and qualitative analyses were followed by the third pattern analysis using the Self-Organising Map Network—SOMNet (machine learning) and Expert-based Collaborative Analysis (EbCA) [40]. The analysis methods are outlined below.

##### Descriptive Analysis

Descriptive statistical analysis (frequency, mean, standard deviation, range) was performed using SPSS version 26 for: contextual (including population) variables; the mechanisms of CM in terms of frequency, duration, and intensity of CM; and percentage of time spent to provide CM actions according to each model. Outcome data—including target of the participant goals according to the ICF, and the phase of recovery achieved—were also examined descriptively.

##### Qualitative Analysis

The descriptive analysis results, combined with other information (e.g., comments about CM and goals, disputes, and complaints qualitative information), provided information for the next stage of analysis which uses machine learning to establish patterns and associations. Some examples include examining the results between health conditions (TBI and SCI) in terms of duration and intensity of CM and which CM actions were performed, and when. This information combined with expert knowledge guides the subsequent SOMNet analysis to examine and ascertain whether there is an association between these variables and the CM actions.

##### Self-Organising Map Network (SOMNet) with Expert-Based Collaborative Analysis (EbCA) [40,41]

The SOMNet is an emerging and novel framework for analysing complex data in diverse application domains such as ecological and health areas [42,43]. Using the SOMNet enables experts to verify a hypothesis, identify hidden information, and elicit tacit knowledge [44]. The SOMNet analysis uses data science techniques (data mining and machine learning) to learn associative relations (patterns) from multiple data sets and develop interactive visual data analytics of the pattern information to obtain insight into the data. Different to other machine learning modelling for classification, a SOM algorithm interprets data in a dataset to find similarities to each other, focusing on their relationship/association [45]. Such a clustering analysis can identify ‘natural’ patterns of the characteristics data around participants, their context, mechanism, and outcomes. These different SOMs are then used as the basic building block for the second stage of the SOMNet analysis in a network fashion of different datasets. The SOMNet itself does not establish cause and effect, rather whether there are associative patterns (or not) between datasets. For this study the SOMNet analysis also involved EbCA and the C-M-O realist theory in the guided processing of the datasets derived (pre, mid, and post processing) [40].

**Figure 2:**
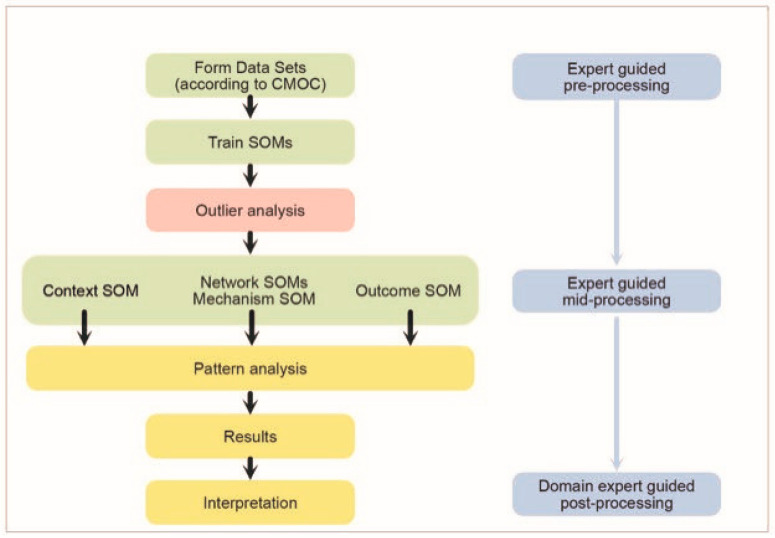
The SOMNet (**left**) and EbCA (**right**) analysis procedure applied to this study.

Refer to Figure 2 for the SOMNet analysis procedure applied to this study.

#### 2.3.5. Interpretation of C-M-O Pattens

Domain expertise and descriptive analysis results were used pre-processing, mid-processing, and post-processing stage of the analysis based on the knowledge discovery at each stage. The domain experts guide the selection of characteristics to be examined for the pattern analysis. For this study, a domain expert is someone with extensive knowledge of CM and the context (systems, services, and interventions) and also the realist theory formulated by the CM experts (C-M-O). The domain expert/s for the analysis was data scientists, the first author and two CM experts from the consensus group. The domain experts considered the focus for the SOMNet analysis in this study as two different datasets (context—mechanism) and (mechanism—outcome) characteristics for each stage of recovery (Recovery, Participation, and Maintenance phases) and each cohort (TBI and SCI).

The visual information of characteristics was presented in the SOMNet for interactive interpretation. The SOMNet provided weights for the association between two datasets, and the weights above a certain point (≥0.8 average) showed a strong association between two characteristics. Only those results that showed an association above the specific weighting point (>0.8 average) are reported in this manuscript.

## 3. Results

The analysis used all sources of knowledge available, and novel analysis methods to identify the links and associations between C-M-O to enable a solid description of what ‘works for whom and when’. We mapped the actions performed by all case managers involved with all participants. With this information, we were able to analyse the patterns of CM actions (and which CM model) associated with enhanced outcomes. The results only relevant to the model of CM are reported in this manuscript.

### 3.1. Description of Context

A total of 107 participants were included in the study. Refer to Table 2 for descriptive information on the context variables. Three participants had sustained both a SCI and TBI. Given the balance of level of spinal injury and brain injury for each of these participants, two were included in the TBI cohort and one in the SCI cohort. There were nine participants who died 5–10 years post injury, eight of whom were people with a TBI. Consequently, the number of participants was reduced (*n* = 98) for the analysis of the Maintaining phase.

### 3.2. Description of Mechanisms

#### 3.2.1. CM Involvement

In the maintaining phase, there were 47% of participants (*n* = 46) who no longer had CM, excluding the deceased participants. Of the remaining participants 23% (*n* = 52) had intermittent CM support, and the remainder (77%) had ongoing CM. Of SCI participants, 56% of participants had CM in the Maintaining phase. In comparison, in Maintaining there were 6 TBI participants receiving intermittent CM and 29 receiving ongoing CM (8.9% and 43.2% respectively), a total of 52%. The mean duration of CM identified was 2454 days (351 weeks or 87.75 months) with a range of 135–3752 days (19–536 weeks or 4.75–134 months). The duration of plans varied across all phases ranging from 3 to 6 months in recovery phase, generally 6–12 months in Participation and Maintaining phases. For those participants who had a CM in the Maintaining phase, the plan if completed, was usually 6–12 months duration.

#### 3.2.2. CM Intensity

**Figure 3:**
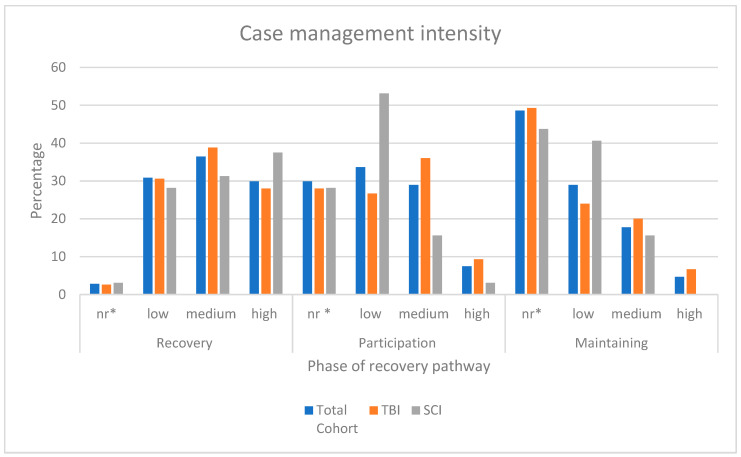
Case management intensity across the three phases in the recovery pathway. Key: * nr. refers to not required, not considered necessary and not performed; low intensity <3 times per month per participant; medium 1–3 times per week, per participant; high intensity (>3 times per week with the participant) [9].

Refers to how frequently CM actions are provided to the same participant in a particular time frame. Figure 3 shows the pattern of the CM intensity for both TBI and SCI cohorts. The graph shows that CM provided to participants with SCI continues at higher rates than for people with TBI, although at a low intensity.

#### 3.2.3. CM Actions Performed

**Figure 4:**
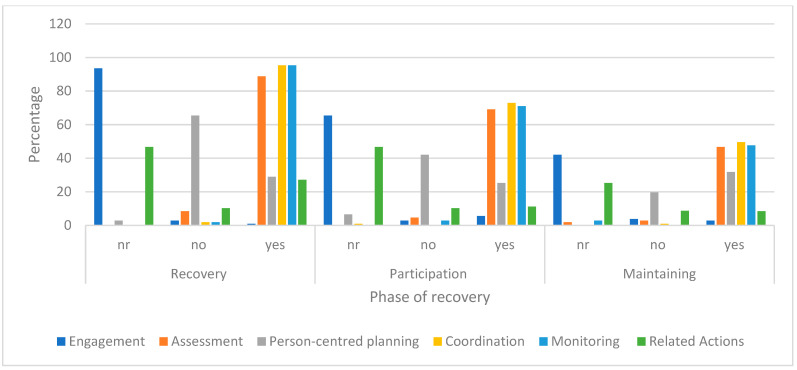
Generalist CM actions across the phases of recovery. Key: The action codes are: nr (not required, not considered necessary and not performed); no—intervention considered necessary but no indication intervention performed; yes—intervention evident and expected.

The following Figure 4 shows the pattern and change in frequency of CM actions performed at each phase for the Generalist model (refer to Table 1 and Supplementary File S3—for full definitions). The graph shows a low percentage of time in person-centred planning or no planning with the person. Percentage of time spent by CM on general assessment (listening and gathering information from other sources) was often absent or limited with most time spent on Generalist model of coordination actions (case consultation, maintaining feedback, and managing documentation).

**Figure 5:**
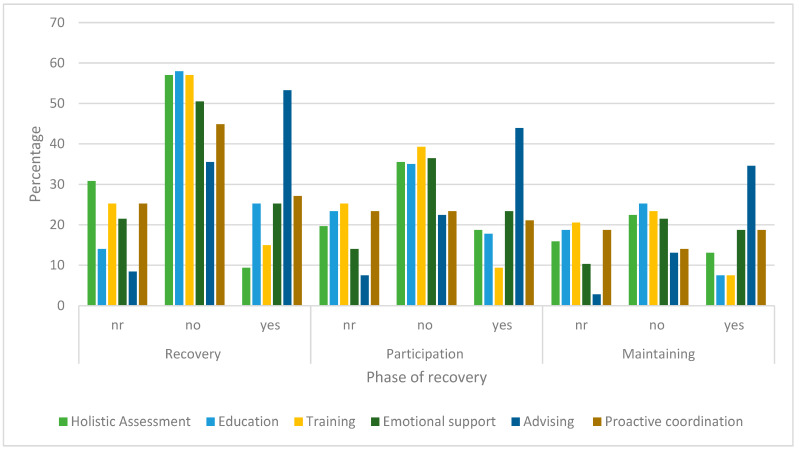
Person-centred CM actions across the periods. The action codes are: nr—not required, not considered necessary and not performed; no—intervention considered necessary but no indication intervention performed; yes—intervention evident and expected; yes, but n/a—intervention performed but not expected nor considered necessary.

In Figure 5, the pattern and change in the frequency CM actions are performed at each phase for the Person-centred model (refer to Table 1 and Supplementary File S3—for full definitions). The graph shows advising (recommending a course of action) is the most frequent action across all three phases.

### 3.3. Description of Outcomes

The major (but not all) outcomes are described below in terms of the target of the participant’s goal according to the domain of the ICF in functioning, the phase of recovery the participant had reached, and when.

#### 3.3.1. Goal Target

The concept behind the participants goal (the target—on what is to be achieved) was mapped to the WHO-ICF classification domains and sub-domains. Table 3 provides the domain one—level classification of the steps per period for each step in the goal and the percentage. There were goal concepts that related to the goals of the case manager (service oriented) and were not participant goals, indicative of the Generalist/broker CM model. These are coded as CM and included in Table 3.

Some of the trends can be summarized as:

- Both the Body Function and CM target goals did not significantly decrease in frequency over the 10 years, indicative of the Generalist CM model.
- A high percent of goals targeted Environmental Factor barriers (e.g., home modifications, provision, and testing of equipment) in the Recovery phase, as might be expected for the study cohort.
- In the Recovery phase only 13.07% of goals or goal steps related to Interpersonal interactions and relationships (d710–d799), with 4.6% of these related to the parent/child relationship. In the Participation phase there were 7.46% of goals or steps related to Interpersonal interactions and relationships, none related to parenting relationship. However, in Year 10 there was a new focus on support and relationships, although only 7.47% were on interpersonal relationships with 2.8% on the parent-child relationship.

#### 3.3.2. The Timelines and Recovery Pathway Phase of the Participant

The timelines and the phase the participant had reached on the recovery pathway was analysed (Refer to 2.3.3-point v. above). The phase along the recovery pathway was an outcome variable (Recovery, Participation, Maintaining) recorded by the file reviewers. At the data collection point of 2 years post the participant’s injury, most participants were still in the Recovery phase (78.5%) with a small number (11.21%) progressing rapidly and thus were in the Maintaining phase. By the 3–5-year post injury data collection point, the participants who were in the Maintaining phase had risen to 35.51% although the majority (43.92%) were considered in Recovery phase with continued progress towards Participation. In the 8–10 year post injury data collection point, many participants were in Maintaining (42.06%), although 21.5% continued in Participation.

### 3.4. Patterns, Associations, and Differences (SOMNet Analysis)

Only the most relevant SOMNet results related to the CM model (Person-centred) are outlined below for each health condition. Refer to Supplementary File S4 for the context, mechanism, and outcome variables. The associations that had a weight >0.8 average only are reported in the Table 4, Table 5, Table 6 and Table 7 below.

#### 3.4.1. Traumatic Brain Injury Cohort SOMNet Results

There are two tables for participants with a TBI. The first details the association between the context and mechanism (CM model) with the second detailing the outcome and mechanism associations.

#### 3.4.2. Spinal Cord Injury SOMNet Results

There are two tables for participants with a SCI. The first details the association between the context and mechanism (CM model) with the second detailing the outcome and mechanism associations.

## 4. Discussion

The Scheme engaged the researchers to undertake a realist evaluation of CM because of the complex needs of Scheme participants and concerns on the quality of CM, considering outcomes and the significant cost. The study demonstrated that a novel research approach can be successful if tools are chosen and adapted to be fit for purpose, to measure and analyse case management models. The tools included: realist CM theory (context-mechanism-outcome); international frameworks and the CMTaxonomy to map and code into categories; retrospective data extracted from file reviews; and novel analysis approaches to scaffold information in a multi-layered data analysis and synthesis guided by CM experts. Learning was the overarching theme in the study design to enable use of the combination of novel tools, to adapt and learn from the data.

The results provide unique insights into what works, for whom and under what circumstances. There are associations and patterns we identified between the cohort of severely injured person’s context, and case management. An overview is provided below.

### 4.1. Case Management Model

For up to 5 years, the full range of CM actions within the Person-centred model are needed for people with SCI and TBI, particularly when the participant’s age at the time of injury is considered [46,47,48]. However, our results show the common model of CM for the study cohort is the Generalist model in the first 2–5 years post injury. We found there was limited Person-centred CM provided, evidenced by the limited holistic assessment actions (the CM measuring outcomes, testing, and observing), Person-centred planning and the infrequency of goals focusing on activities and participation.

In the critical 2–5 years after a severe injury, the case manager typically did not collaboratively plan with the person in terms of individualised goal and priority setting, responsibilities, and service provision. This is reinforced by the fact that over time the person’s goals to engage in occupationally oriented pursuits and life roles (including voluntary work, work preparation, employment, education/study, purposeful community-based activities) reduced—rather than increased—as physical recovery improved, stabilised, or was sustained. Participant goals were consistently targeting body function and structure over 10 years rather than participation and resuming life roles. Whilst a focus on post-acute recovery of body functioning is expected in the first 1–2 years, thereafter it is critical that case managers support the participant to focus on resuming activities and participation in life roles [14,46].

Severe injury with ongoing impairments typically poses significant challenges to interpersonal relationships and the person’s informal (unpaid) supports from family and friends [46,47,49]. Yet, our study results demonstrate a gap in goals related to interpersonal and personal relationships. The goal target of interpersonal or personal relationships was limited and decreased rather than increased over the 10-year period.

Case managers need to adopt person-centred planning and greater attention to issues, barriers, and facilitators to interpersonal and personal relationships including parent/child relationships (where relevant) for people who have complex and long-term health conditions. Our results show that when a Person-centred CM model was adopted, the case manager actions were associated with the enhanced progress and outcomes for the person, particularly for the first two years post injury. This confirms the value of Person-centred CM model compared to the Generalist model.

### 4.2. Duration of Case Management

Case management was provided to the participants for an average of 7 years (range 5 months to 10 years). As expected, the CM actions decreased over time in line with the phase of recovery. However, participants with SCI had a higher rate of continued CM for 10 years compared to those with TBI. This is despite the significant differences of health condition impacts of TBI across multiple health systems (cognitive, neurological, physical, behavioural, and emotional) compared to SCI (neurological—sensory and motor impairment) and the complexity of these impairments on functioning. Our results confirmed that participants with TBI (compared to those with SCI) live in less stable rental accommodation and are less likely to be in paid employment at 10 years. These additional contextual barriers to reaching Participation and Maintaining phases for participants with TBI suggest a continuing need for Person-centred CM to provide the necessary coordination and supports for the participant with TBI to obtain more stable accommodation and achieve purposeful activity such as employment.

Our study results suggest that CM may be unnecessarily involved with some participants with SCI in the Maintaining life phase. The CM actions in these instances were ‘related coordination actions’ (coordination-related actions performed by the CM that were not expected to be done nor considered necessary for a CM to perform) in the Recovery phase and in Year 10. At these times, the participant was in the Maintaining phase. Potentially the participants with SCI had the capacity to perform these actions themselves by year 10, or that a routine support service could assist at a lower cost than a health professional trained case manager.

### 4.3. Case Management Actions and Context

Person-centred case management involves changing what actions are performed with whom, when according to the person’s context and needs. The CM actions are discussed below with respect to the two health conditions.

Traumatic brain injury: A range of characteristics of the participant with a TBI were associated with a higher intensity and Person-centred CM model up until 5 years post injury. These were: the participants’ age at the time of injury; whether they were married; completion of secondary education; vocationally or tertiary training and were perceived to hold a positive attitude towards their recovery (personal factor is a facilitator). Typically, this cohort involved people less than 25 years old at time of injury. This could be due to their age, Recovery phase, and the dynamics of CM working with a couple (possibly children) and resuming life roles. For those living with their spouse or partner the level of coordination provided was less. People who were single or divorced at the time of the injury typically had a CM involved for 5 years, who performed advising and coordination actions. After 5 years, participants who were single or living alone, the costs were higher and the key CM actions were advising and coordination. However, the level of functioning was not associated with CM actions until year 10, when there was an association because of accident-related chronic pain and/or mental health concerns.

Spinal cord injury: The participant’s relationship status at the time of the accident was associated with CM actions. However, the set of actions for those who were single varies to those who were married. For example, if single there was more education provided by the CM and later if in a relationship there was an association with CM coordination and assessment actions. People with SCI and who were vocationally trained at the time of the injury were associated with a suite of CM actions, particularly from the Participation phase. The reasons could be related to the participants age, previous work and focus an occupational status or perhaps changes in other areas of their life (e.g., accommodation). For the participants with SCI there was an unexpected pattern with CM actions and the severity of the injury. In the Recovery phase, the association of CM actions remains similar between cervical and thoracic injuries (incomplete). However, in the Participation phase, the range of CM actions increased the lower the level of motor spinal cord injury and the less severe the injury. It is not clear why all Person-centred CM actions are performed with participants with the least severe SCI (level and sensory/motor).

### 4.4. Case Management and Outcomes

For participants with TBI, there was an association with Person-centred CM model actions and the participant moving forward in their Recovery phase post injury. The CM actions contribute to the person reaching the phases of Participation and Maintaining. The CM intensity and actions that particularly influence the participants progress are advising, emotional support, and coordination. By year 10, other contextual factors also contribute including stable accommodation, care support services, informal supports, and continued intermittent CM for better outcomes. For participants with SCI the Person-centred CM model also contributed to most of the participants reaching the Maintaining phase by the two years. However, the data show that, surprisingly, few were working or studying and so had not established an occupational participation focus, even though a case manager was involved.

### 4.5. Limitations

Data collection from retrospective file reviews was limited by the information available. Although every step to minimise errors was taken, judgments made by file reviewers may be less reliable than concurrent data collection by the case managers. The sample size restricted our ability to quantify cause–effect associations. Nevertheless, our findings generate new hypotheses for future investigations on models of CM. The realist theory framework informed what variables to look at first for the SOMNet analysis. Due to time and resource constraints, we were not able to look at SOMNet for context and outcomes. As realist evaluation is iterative, there are numerous other relationships that could be explored without any further data extraction. This may be a consideration for further study.

Future research may build on the use of novel approaches and tools to ‘unpack’ and appraise the various CM models to strengthen an evidence base for best practice, policy, CM education, service planning, and management. Data collection concurrent to service provision would significantly facilitate future research. Comparisons of CM provided to people with other health conditions would also assist to examine the quality and model of CM.

## 5. Conclusions

No similar study examining different CM models using a realist evaluation and novel analysis methods was identified. This study provides unique insights on the best practice model of Person-centred CM for people with complex health conditions and the association with their recovery outcomes. The Person-centred CM involves the actions (mechanisms) of planning with the person to develop and enact individualised goals, proactive coordination and collaboration, monitoring, education, training, and emotional and motivational support as needed. The learnings can be applied to future CM quality appraisal, service policy, planning, and management.

## Figures and Tables

**Table 1:** The model of case management mapped to the case management taxonomy actions (mechanisms) *.

Case Management Actions and Definitions (Refer to the CMTaxonomy Intervention Tree)	Generalist Model	Person-Centred Model
***Engagement^*:** Establish, develop, and maintain a relationship with the client.	X	X
***Assessment:*** through listening and gathering information from other sources (e.g., reports from therapists, doctors, teachers, vocational professionals)	X	X
***Holistic assessment*:** Assessment conducted by the case manager through observation of functioning, testing, and measurement of outcomes		X
***Person-centred planning*:** Supporting the person to develop their individualised plan including setting goals and identify support needs as well, as therapy and rehabilitation related goals		X
***Advising:*** Recommending a course to be followed, encourage change in functioning, environment, and attitude or behaviour to meet health needs and goals, and reduce risk		X
***Coordination:*** Case consultation, maintaining feedback, managing documentation	X	X
***Proactive coordination:*** Including navigation, facilitation, advocacy for services, and extensive collaboration		X
***Education:*** Providing structured information to the person, and/or their family, other stakeholders in a manner conducive to improve knowledge about the persons’ condition, treatment, functioning, or strategies		X
***Training*** and skill development: Teaching, enhancing, or developing skills through context-specific practice to stakeholders		X
***Emotional****(and Motivational) **support:*** Providing the person (and others as appropriate) with comfort, empathy, or motivational support		X
***Monitoring:*** Continuous acquisition of information to evaluate the person’s situation to determine their progress, anticipate problems	X	X
***Related actions:*** Actions that are less frequently performed to facilitate coordination (practical support and performing tasks—e.g., making a medical appointment).	X	X

* Abbreviated definitions from the CMTaxonomy–refer to the Glossary in Supplementary File S3 for full definitions. ^ These terms in ***italics and bolded*** are used to describe the actions (mechanism) in the two CM models throughout the manuscript.

**Table 2:** Key study cohort context variables.

Variable	N	%	Mean	Range	SD
Health Condition					
TBI	75	70	n/a *	n/a	n/a
SCI	32	30	n/a	n/a	n/a
Gender					
Male	77	72	n/a	n/a	n/a
Female	39	28	n/a	n/a	n/a
Age at injury	107	n/a	32.2	8–96 years	20.16
Age at time of study	107	n/a	45.8	19–106 years	20.16
Personal relationship					
Pre-injury	58	54	n/a	n/a	n/a
Year 2	53	49.4	n/a	n/a	n/a
Year 5	49	45.9	n/a	n/a	n/a
Year 10	39	36.4	n/a	n/a	n/a

* n/a—not applicable.

**Table 3:** Percent and patterns of the most frequent ICF domains for participant goals mapped per period.

Phase Post Injury	Recovery Phase		Participation Phase		Maintenance Phase	
ICF Domain	Number	%	Number	%	Number	%
**Body functions and structures**	69	16	30	14.4	25	15.7
**Most frequent sub-domain**	Neuro-musculoskeletal movement related, mental functions, sensory functions, and pain		Structures of the nervous system (e.g., parasympathetic nervous system)		No frequency pattern observed	
**Activities and participation**	206	48	135	65	104	66
**Most frequent sub-domain**	Major life areas (i.e., education, work, managing money), self-care (looking after one’s health), mobility, general tasks and demands (e.g., undertaking multiple tasks, carrying out daily routine and handling stress)		Self-care (with a high frequency of maintaining one’s health ^1^), major life areas (education and employment), mobility, general tasks, and demands		Major life areas (acquiring, keeping and maintaining paid employment), self-care (changed focus to—diet and fitness and managing one’s health), community, social and civic life (recreation and leisure)	
**Environment**	114	26	18	8.6	19	12
**Most frequent sub-domain**	Products and technology (specifically equipment activities of daily living)		Products and technology (for personal use)		Products and technology (for personal use)	
**Case management**	42	10	24	12	10	6.3

^1^ Defined in the ICF as ‘being aware of the need and doing what is required to look after one’s health, both to respond to risks to health and to prevent ill-health, such as seeking professional assistance, following medical and other health advice and avoiding risks to health such as physical injury, communicable diseases, drug-taking and sexual health’.

**Table 4:** SOMNet Results TBI cohort association of context with mechanism variables (weighting >0.8).

Context Variable	Association with Mechanism (CM Actions and Intensity)
Age	In the first 5 years following injury, a person’s age at the time of the injury was associated with CM actions. In this cohort, the mean age of participants was 25 years old when injured in motor vehicle crashes. All Person-centred CM actions included in the analysis were used more frequently (holistic assessment, emotional and motivational support, education, training and skill development, advising, and proactive coordination).
Functioning impairment	The level of functioning and impairment and accident-related chronic pain were not associated with CM actions until year 10, when chronic pain and mental health were associated with the actions of the case manager.
Level of education	By year 5, there was an association of CM actions for those people who had completed secondary and tertiary education preinjury with all Person-centred CM actions, but not for those who completed vocational education. The reason could be due to the participant resuming life roles as most participants were in the Participation phase by year 5. Continuing up to the phase of maintaining health and wellbeing, being married and/or vocationally or tertiary educated at the time of accident were associated with CM actions of person-centred coordination and assessment.
Participant attitude	The participant’s faciliatory/positive attitude towards their own recovery had an association with the CM advising and coordination (but did not increase the costs). The association was not there for participants if they had a negative attitude towards their recovery.
Living with spouse/partner	Up to year 5, there was a strong association with CM action proactive coordination (navigating, facilitating, advocating, and collaboration) and the CM intensity was strongly associated when the participant was living with their spouse. The data showed that proactive coordination actions were less frequently performed when the participant lived with their spouse/partner rather than more. When the participant had a spouse/partner involved, the intensity was higher. Quite possibly the higher intensity is because of the complexity and dynamics of working with a couple in a household, and CM related to two people (there may also be children in the household).
Living alone	Intensity was also associated with people who were single and living alone at any phase in the recovery pathway.

**Table 5:** SOMNet Results TBI cohort association of outcomes with mechanism variables (weighting >0.8).

Outcome Variable	Association with Mechanism (CM Actions and Intensity)
**Progress on the pathway** (progress along the recovery pathway from Recovery to Participation to Maintaining)	At all three phases in the recovery pathway (Recovery, Participation, and Maintaining), there were strong associations with all Person-centred CM actions. In particular, the Person-centred CM actions contributed to the participant reaching the Participation phase by the 2 years post injury period (78% of participants). This was reinforced by the association of CM intensity for participants who had reached either the Participation or Maintaining phases, in particular the CM action of advising.
**Participation phase**	In the Participation phase, there were associations with CM intensity and, CM actions of education, emotional support, proactive coordination (but not advising). The results consistently show that Person-centred CM model supports participants to progress along the recovery pathway particularly if the CM is involved in the early stages following their injury.
**Maintaining health and wellbeing phase**	When a participant continued to have CM involvement in year 10, all participants were in either the Participation or Maintaining phases. CM intensity was high for those in Participation and less intensity if in Maintaining. In the Maintaining phase, there was no association with continuous CM (versus intermittent CM) on other outcome variables measured in the study. The result suggests that by year 10 continuous CM (compared to intermittent CM) is not associated with other measured outcomes (e.g., further education).
	There was an association between holistic assessment, stable accommodation (> 2 years) and intermittent CM. In these circumstances, the participant reached the Maintaining health and wellbeing phase.
	The intensity of case management, the length of time living in the current home, and intermittent CM were associated with the phase post injury (Participation and Maintaining).

**Table 6:** SOMNet Results SCI cohort association of context with mechanism variables (weighting >0.8 average).

Context Variable	Association with Mechanism (CM Actions and Intensity)
Level of spinal cord injury	In the first 2 years, following injury the CM actions of holistic assessment, advising, proactive coordination was associated with participants who had sustained a cervical SCI. Whereas with thoracic SCI there was a different configuration with holistic assessment, emotional support, and advising. There were no CM action associations with the lumbar SCI in this period. Over the next 2–5-year period, there was still an association with CM actions of holistic assessment and proactive coordination for people with cervical SCI, with thoracic SCI, American Spinal Cord Association Impairment Scale (ASIA) ASIA B and ASIA C participants having an additional association with CM action of education. Participants with lumbar SCI was associated with all six Person-centred CM actions. In the Participation phase for ASIA A there was only an association with proactive coordination, whereas ASIA D had an association with all six CM actions.
Motor and sensory function	In the first 2 years after injury, there was no association with the severity of motor and sensory spinal injury ASIA A ^1^ (complete no motor and sensory function below the level of injury), although there was an association with ASIA B (incomplete some sensory but no motor function) but only for CM education. For ASIA C (some limited motor function) the actions of assessment, emotional support, advising, and coordination were all significantly associated. However, there was no association with ASIA D (some motor weakness).
Functioning	For the first 5 years post injury, accident related-pain was associated with all Person-centred CM actions, and where there were accident-related mental health issues, the CM actions of assessment and proactive coordination.
Level of education	If the participant had vocational or tertiary education prior to their injury, there was an association with most Person-centred CM actions in the Participation phase.
Participant attitude	Participants with a positive attitude towards their recovery were associated with all Person-centred CM actions up until the Participation phase. At the 5-year period, if the participants’ attitude towards their recovery was negative, there was an association with CM actions of education and proactive coordination.
Living with spouse/partner	Holistic assessment action was associated with participants who were married living with their spouse. There was an even stronger association with holistic assessment for participants who were divorced. Case management costs were associated with who the participants lived with (their spouse, single, divorced, or living with their family of origin). CM proactive coordination was associated with Participation phase, but strongly associated with people who were divorced in the Recovery phase and less so people married and living with their spouse.
Living alone	Being single at the time of the accident was associated with all the Person-centred CM actions in the Participation phase. Participants who were single post injury were also associated CM actions of education, training, and skill development, advising in the first Recovery phase.

^1^https://www.physio-pedia.com/American_Spinal_Cord_Injury_Association_(ASIA)_Impairment_Scale (accessed on 13 December 2018).

**Table 7:** SOMNet Results SCI cohort association of outcomes with mechanism variables (weighting >0.8 average).

Outcome Variable	Association with Mechanism (CM Actions and Intensity)
**Progress on the Pathway** (progress along the recovery pathway from Recovery to Participation to Maintaining)	All participants considered to be in the Maintaining phase in Year 10 had a case manager involved, be it ongoing or intermittent. The most frequent CM action was proactive coordination (notably not coordination). This suggests a dependency of the participant on the CM.
**Recovery phase**	CM education, training, and skill development, advising in Recovery phase were associated with the target of the participant’s goals
**Participation phase**	CM intensity in year 5 was associated with the Participation phase. In the Participation phase there was high frequency of all CM actions with the most common CM action was proactive coordination. Holistic assessment action was associated with the participant being in the Participation phase. Proactive coordination was associated with goal rating in the Participation phase.
**Maintaining health and wellbeing phase**	There was an association with CM costs and goal rating, if the participant had reached the Maintaining life phase by year 5. This suggests that the involvement of the case manager is associated with the participant moving further along the recovery pathway within 5 years post injury.
	For participants with SCI, most were in Maintaining phase by Year 5, with associations of all Person-centred CM actions and the plan period. However, only 6 of the 18 participants in the Maintaining phase were working, none were studying (including work preparation and voluntary work). Less than half had therefore established their occupational focus. By Year 10, there were no additional participants employed and only one additional participant had retired, and one was studying.
	CM action advising and coordination were associated with the Maintaining phase

## Data Availability

The data presented in this study are not publicly available. Ethical clearance for sharing individual level data was not obtained.

## Supplementary Materials

The following supporting information can be downloaded at: https://www.mdpi.com/article/10.3390/ijerph20054362/s1, File S1: Data extracted; File S2: CMTaxonomy Intervention tree; File S3: CMTaxonomy Service tree; File S4: CMTaxonomy glossary; File S5: Framework and categories. Ref. [50] is cited in Supplementary Materials.
