# Supplementary material for: A Realist Evaluation of Case Management Models for People with Complex Health Conditions Using Novel Methods and Tools—What Works, for Whom, and under What Circumstances?
Source: Int J Environ Res Public Health. 2023 Feb 28;20(5):4362. doi: 10.3390/ijerph20054362 (PMC10002263; doi:10.3390/ijerph20054362)
Supplement: Supplementary file 1 [file ijerph-20-04362-s001.zip › File S1 Data extracted .pdf]

Supplementary File S1 Data extracted on the following variables in the file reviews per C-M-O-C

Context	Mechanisms	Outcomes
• Gender<br>• Date of accident<br>• Injury and diagnosis related scores on standardised measures (performed by the treating health practitioners) *<br>• Age at time of accident and at year 2,5,10<br>• Time between injury date and (interim) participation in the LTCS (and reason for delay)<br>• Preinjury medical history<br>• Preinjury co-morbidities<br>• Accident-related co-morbidities (mental health, chronic pain, drug and alcohol problem),<br>• Language spoken at home,<br>• Role in the accident (at fault/not at fault)<br>• Education<br>• Personal factor (barrier of facilitator)<br>• Complaints and disputes on file for participant to LTCS on treatment, rehabilitation, and care (data obtained from different section in LTCS)<br>• Date of death and cause (where applicable)<br>• Criminal history,<br>• Pre- injury personal and social indicators (Drugs and alcohol, mental health)<br>• Under Guardianship and financial management order across 2,5,10 years<br>• Challenging behaviours<br>• Claims status<br>• Attitude of the family towards the participant year 5 and 10 years<br>• Family issues identified in pre-injury at 2, 5, 10 years	• Informal supports<br>CM period<br>• CM intensity (low, medium high defined in CMTaxonomy)<br>• CM involvement at year 10<br>• CM whether continuous, intermittent, ongoing<br>• CM actions (interventions)<br>• Cost of attendant care<br>• Cost of treatment and rehabilitation<br>• Cost of case management<br>• Source of income<br>• Cultural factors perceived to influence (reviewer judgment made after reviewing content of >5 years CM and relevant reports)<br>• Informal supports beyond usual family expectations<br>• Change in case managers over time<br>• Case management date of closure<br>• Case management actions for years 2,5,10 (engage, Assess 1, Assess 2, Plan, Education, training and skill development, planning, emotional and motivational support, advising, coordinate 1, coordinate 2, monitoring, related actions of coordination)<br>• CM Comments<br>• Service codes and costs for treatment and rehabilitation, medical services, attendant care support, case management (codes identified through invoice codes)	• Driving status<br>• Recovery phase (pathway from My Plan)<br>• Goal outcome rating (year 2,5,9) for 3 goals<br>• Vocational status<br>• Relationship status (2,5,9)<br>• Living situation (with whom)<br>• Years in current home (year10)<br>• Type of accommodation<br>• Postcode (year 2 and 9)<br>• Perceived participant adjustment 2 ,5 ,10 years<br>• Family breakdown by 10 years<br>• World Health Organization Quality of Life measure (WHOQoL) year 10<br>• Case management involvement year 10<br>• Length of case management plan years 2,5,10<br>• goal concept mapped to an ICF code (for each 2-, 5- and 10-year period)

*examples of standardised measures include the Post traumatic Amnesia (PTA), Functional Independence Measure (FIM), Spinal Cord injury Measure (SCIM), Care and Needs Scale (CANS)
