# Supplementary material for: A Realist Evaluation of Case Management Models for People with Complex Health Conditions Using Novel Methods and Tools—What Works, for Whom, and under What Circumstances?
Source: Int J Environ Res Public Health. 2023 Feb 28;20(5):4362. doi: 10.3390/ijerph20054362 (PMC10002263; doi:10.3390/ijerph20054362)
Supplement: Supplementary file 1 [file ijerph-20-04362-s001.zip › File S4 CMTaxonomy glossary.pdf]

GLOSSARY - case management taxonomy

Acceptance of referral - Clarifying the match (or not) of the purpose of the client's referral, their context and the funding system with the case manager's expertise, capacity and availability; seeking an overview of the client, their location and possible needs including jurisdiction, related policies and systems e.g. Lifetime Care & Support Scheme, Workers Compensation, Transport Accident Commission, National Disability Insurance Scheme.

Action - A deed, which is done by an actor to a target during a healthcare intervention (WHO ICHI Development Project, 2013)

Acute – Refers to the service which provides care to clients who are experiencing a period of crisis or deterioration in physical, mental state, behaviour or social functioning related to their health condition and that the purpose of the service is to alleviate the deterioration (modified from (Salvador-Carulla et al., 2011)).

Advocating - Mediation or pleading in favour of a client including lobbying to achieve access for the client to existing resources or services. [modified (Fortune, Almborg, Cumeralato, & Best, 2014)

Advising - Recommending a course of action to be followed, to encourage a change of functioning, environment, attitude or behaviour in relation to health, goals or risks. (Fortune et al., 2014) Excludes: counselling and psychotherapy

Ascertain capacity for decision-making – capacity for decision making around issues such as finances, legal and quasi-legal matters

Bridging – Building partnerships and coalitions between groups or organisations

Care - The support, services and assistance provided to a client. Includes: formal and informal supports, aids and equipment, and care related products

Case Manager – A skilled person who provides the case management interventions to the client.

Client – A client is the individual person who is the user of the case management intervention.

Client support –

> **Task performed by case manager** - Performing a task on behalf of the client (Fortune et al., 2014) e.g. making a medical appointment
> **Practical support** - Providing practice assistance or guidance to facilitate activities or participation (Fortune et al., 2014) e.g. attending a medical appointment with the client

Collaboration - The action of working and cooperating with one another

Community-based case management - Community-based case management is a multidimensional and collaborative process. It involves a set of interventions for assessment, planning, coordinating and review of the options and services required to meet the client's health-related needs, and support them to reach their goals related to participation in life roles.

Consultation - Discussion with stakeholders to plan, improve and promote teamwork and achieve the agreed goals. Includes: meeting of multiple parties providing health service delivery or supports

Context - The factors that together constitute the complete circumstances of the person's life including the environmental factors that are external and extrinsic (e.g. products and technology, natural and human-made environment, support and relationships, attitudes, services, systems and policies) and the personal factors that relate to the individual' particular background and living (e.g. age, gender, motivation, habits, upbringing, coping styles, social background). (World Health Organization (WHO), 2001)

Coordination - Navigating and facilitating the access, management and cohesion of services and supports for the client**.**

Decision-making supports - Supports and ways used to enable the client to make their own decision. Some examples of supports to enhance access to information and understanding may be plain English explanations, communication aids, language translators. Examples of supports for decision making and choice may be the use of pictures, diagrams or demonstrations to better present options and anticipated outcomes(Lifetime Care & Support Authority (LTCSA), 2014).

Disability - Umbrella term for impairments, activity limitations and participation restrictions; it denotes the negative aspects of the interaction between and individual (with a health condition) and that individual's context (environmental and personal factors) (World Health Organization (WHO), 2001).

Education - Providing structured information to client and stakeholders in a manner conducive to improve knowledge about matters relevant to the client's health condition, medical, or rehabilitation treatment, functioning, situation or strategies (Fortune et al., 2014).

Emotional and motivational support - Providing the client (family and others as appropriate) with comfort, empathy or motivational support
Includes: supportive communication (without using theory based methods) to find strategies to solve or alleviate difficulties arising from their daily demands of life and situation (Fortune et al., 2014); assisting, encouraging and reinforcing the client (and family as appropriate) to build independence, make decisions, exercise choice and responsibilities, take actions, and support the client's and family's adjustment to changed circumstances

Engagement - Establish, develop and maintain a relationship with the client.

Establish partnerships - Collaborating with the client, family and other stakeholders to establish a relationship, and develop and maintain a partnership. Includes:

- Obtaining consent
- Identifying other key people to engage
- Establishing and managing expectations of client and stakeholders
- Education on the role of case manager

Expectations - A strong belief that something is true or will be the case

Facilitating - Making the process easier, identify gaps, anticipate problems, help remove or negotiate barriers, and promote safe and effective connections to services and appropriate use of resources.

Facilitation and support of client planning (to the extent possible or chosen by client) Includes:

- Identifying their goals and priorities
- Planning steps and actions
- Identifying resources, supports and barriers
- Identifying longer terms needs for support and resources (e.g. when case management needs to be re-instated, care, informal supports, network)
- Formalising a process for monitoring maintenance of outcomes achieved
- Reviewing for success, strategies and safeguards: weighing up the potential benefits, lessons learnt and what is important, the facilitators and barriers, while respecting and supporting client choice
- Identifying client informed decisions, including plans for safeguards and responsibilities
- Finalisation of plan: reconsidering and revising plan with client considering all information from client, scientific evidence and facts, professional experience, shared perspective and practical considerations.
  Includes: managing documentation and recording information about an individual, group or environment (WHO ICHI Development Project, 2013)

Family - A family is two or more persons, who are related by blood, marriage (registered or de facto), adoption, step or fostering, [modified (Australian Bureau of Statistics (ABS), 2011)

Formal supports - A task or work done by someone for another as a job or duty or task (paid). The support is usually organised or structured. Government, non-profit organisations and the for-profit sector usually provide the services. The term used to describe a person employed to perform the task may be support worker, attendant care worker, personal care assistant, domestic assistant, maintenance assistant or care worker. Includes:

- Community support and independent living support services to provide assistance with self-care, household duties, mobility and moving around the community
- residential and respite support services, classroom assistance in education or personal support in the workplace, communication supports, supported decision making, advocacy, peer support, assistance animals, or periodic

allied health services to maintain body function e.g. podiatrist (Lifetime Care & Support Authority (LTCSA), 2014).

Functioning - Is an umbrella term for body functions, body structures, activities and participation. It denotes the positive aspects of the interaction between the individual (with a health condition) and that individual's context (environmental and personal factors) (World Health Organization (WHO), 2001).

Gathering information from other sources – Includes:

- Other reports and assessments e.g. medical history
- Past and current activities and participation
- Identifying barriers and facilitators in the client's context e.g. client's, family and community strengths
- Identifying areas of unmet need of client.

Goal and goal setting - A goal is what the client aspires to achieve, the object of their ambition or effort: a desired end or result. Goal setting refers to the process of identifying the client's goals, establishing the action plan and then monitoring the progress towards achieving the goal (Brain Injury Rehabilitation Directorate (BIRD), 2014).

Health - Health refers to the level of functioning within a given health related domain (e.g. body structures, activities, participation) (World Health Organization (WHO), 2001).

Health condition – Umbrella term for a disease (acute or chronic), disorder, injury or trauma; a health condition may include other circumstances such as pregnancy, ageing, stress, congenital anomaly, or genetic predisposition (coded using the International Classification of Diseases - ICD)(World Health Organization (WHO), 2001).

Health related domains - Body functions, body structures, activities and participation, life areas, internal influences on functioning and disability (personal factors), external influences on functioning and disability (environmental factors)(World Health Organization (WHO), 2001).

Holistic assessment - Evaluating the client's health condition, functioning, environment, behaviour, situation or need for intervention(Fortune et al., 2014); in order to develop a comprehensive understanding of them, their perspective, and what is important to them. Includes: their strengths, capacity, performance and needs across domains in relation to health, participation in key life areas (education, work, social, cultural and civic life), well-being and the contextual barriers and facilitators (Lukersmith, Fernandez, Millington, & Salvador-Carulla, 2015) ; assessment for the purpose of identifying appropriate intervention(s) and planning interventions (Fortune et al., 2014).Excludes: monitoring

Informal supports - Resources (goods, services or a person who performs a task or work) that are inbuilt or purposively developed in the person's environment and

enhance quality and security of life. Informal supports support the person when there is a need and may be reciprocated with different goods, services or tasks (unpaid). Someone in the person's network including family member, friends (family or friend might be referred to as a carer) or members of a community (unpaid) often provide the informal supports. The support is typically "ordinary" and less formal and may be ad hoc. Participation and membership to clubs, interest groups, organisations, classes/education, volunteer work programs and other civic activities often result in a network and a range of supports that are informal. A person's network can provide the opportunity for support (Lifetime Care & Support Authority (LTCSA), 2014).

Intensity – The capacity of the service to provide community case management interventions on different days, related to the same client. (modified (Salvador-Carulla et al., 2011))

Intervention - An activity performed for, with or on behalf of a client or a population whose purpose is to improve, assess, or modify, health, functioning or health conditions (WHO ICHI Development Project, 2013).

Linking – Linking client with appropriate supports and agencies e.g. referring - the action of sending the client to see another person or place for consultation, review or further action, help or advice.

Listen - Listening to understand the person's perspective.

Maintaining feedback - Communicating, giving to and receiving information from stakeholders

Managing - Delegating, managing or monitoring services or supports on behalf of client (defined in the action Brokerage (Fortune et al., 2014) modified).

Managing documentation and information between stakeholders - Recording information about an individual, group or environment e.g. case conference decisions, progress reports, concerns and barriers, request for services, referral, linkage and liaison with service providers, agencies and clients.

Means – describes the processes and methods by which the action is carried out e.g. approach, technique, method or sample (WHO ICHI Development Project, 2013)

Measurement of outcomes - Quantitative determination of characteristics of body parts, functioning or environmental factors which results in a continuous variable (WHO ICHI Development Project, 2013). Includes: Standardised assessment, observation and client self-evaluation

Mobile – Contact with the client occurs in a range of settings including the client's home as judged appropriate by the case manager and client (Salvador-Carulla et al., 2011).

Monitoring - Continuous acquisition of information to evaluate the client's health condition, functioning, environment, behaviour or situation over a defined period

(Fortune et al., 2014) in order to be able to determine their progress, anticipate or identify problems, additional goals or activities and modify plan and services as appropriate.

Non-acute – Refers to the service which provides clients with continuing care including regular contact with the case manager, which may be long term if required. Continuing care services may also provide acute care on a regular basis but this forms less than 20% of the usual activities of the service (modified (Salvador-Carulla et al., 2011)).

Navigating - Finding the most appropriate pathway through systems, services, resources and supports for the client given their context.

Observation – Watching and seeing to acquire information, and understand the client's situation, functioning, environment and behaviour (modified (WHO ICHI Development Project, 2013))

Person centred - A perspective and a way to discover and understand what people want and what they need. A person-centred approach in planning for community participation emphasises seeing the participant as himself or herself and adopting practices, which pro-actively considers the person's own context and situation. This means looking beyond the person's health condition or injury, and the services each organisation provides. Person-centred practitioners holistically look towards the person's aspirations and goals for participation in life, their needs and preferences as well as actively supporting them to be involved in planning for supports (Lifetime Care & Support Authority (LTCSA), 2014).

Personal factors - Factors that relate to the individual's particular background life and living and that are not part of the features of the health condition such as age, race, gender, social status, lifestyle, habits, upbringing, coping styles, social background, education, past and current life experiences (World Health Organization (WHO), 2001).

Planning - Supporting the client to develop their individualised plan including setting goals and priorities, actions, responsibilities to achieve the goals and identify the supports needed (services and resources).

Planning long term supports - Identifying, promoting and supporting the client's ownership and independence for management and coordination of their activities in key life areas, to resolve problems, in order to reduce or cease their need for paid case management (to the extent possible and including the family or significant others). Includes:

- Identifying timing and manner for case management withdrawal
- Supporting client to perform case management activities for themselves including self advocacy.

Preparation - Performing initial work to promote good practice, management and success e.g. discuss options with service providers, or set up a trial for an upcoming plan.

Referral - The action of sending someone to see another person or place for consultation, review or further action, help or advice.

Self-advocacy - Refers to the client being able to lobby or plead on their own behalf.

Service - An activity, help or aid for another person.

Stakeholders - Any person or organisation interested and/or involved in the client's circumstances. Includes: client, family, case manager, informal support persons, funder & service providers (e.g. therapists, teachers, attendant care workers, volunteers, unpaid carers, community support persons)

Support - A task, services, goods or work done by someone for another. Formal support is a paid job or duty or task, whereas informal support is unpaid (Lifetime Care & Support Authority (LTCSA), 2014).

Target – The entities on which the action is carried out e.g. human function, the person, their activities and participation, behaviour or environmental factors (WHO ICHI Development Project, 2013)

Test - Evaluating the client's health condition, their functioning, environment, behaviour or situation using an assessment instrument (e.g. manual-based questionnaire, rating scale, semi-structured interview, standardized instrument) or screening tool (Fortune et al., 2014).

Training and skills development - Teaching, enhancing or developing skills through context-specific practice (Fortune et al., 2014) to stakeholders. Includes: providing information or reinforcing training strategies developed by others for skill development e.g. memory or anger management strategies

References

Australian Bureau of Statistics (ABS). (2011). *Census Dictionary* Canberra, ACT: ABS

Brain Injury Rehabilitation Directorate (BIRD). (2014). *Goal training* Sydney ACI

Fortune, N, Almborg, A, Cumeralato, M, & Best, L (2014). Revised action defnitions for ICHI Alpha 3 - 22 April.

Lifetime Care & Support Authority (LTCSA). (2014). *My Plan Manual (draft)* Sydney LTCSA

Lukersmith, S, Fernandez, A , Millington, M, & Salvador-Carulla, L (2015). A preliminary taxonomy of community-based case management in brain injury - the BICM-PT

Salvador-Carulla, L, Dimitrov, H, Weber, G, McDAid, D, Venner, B, Sprah, L, . . . (eds), for DESDE-LTC Group (Eds.). (2011). *DESDE-LTC: Evaluation and classification of services for long term care in Europe,* . Spain

WHO ICHI Development Project. (2013). *International Classification of Health Interventions (ICHI) Alpha 2 version*

World Health Organization (WHO). (2001). *International Classification of Functioning, Disability and Health (ICF),* . Geneva WHO
