# Supplementary material for: A Realist Evaluation of Case Management Models for People with Complex Health Conditions Using Novel Methods and Tools—What Works, for Whom, and under What Circumstances?
Source: Int J Environ Res Public Health. 2023 Feb 28;20(5):4362. doi: 10.3390/ijerph20054362 (PMC10002263; doi:10.3390/ijerph20054362)
Supplement: Supplementary file 1 [file ijerph-20-04362-s001.zip › File S5 Framework and categories.pdf]

Supplementary File S5 Framework and categories for specific variables.

Variable	Informing framework	Categories and description
Perceived participant adjustment (years 2,5,10)	World Health Organization ICF (environmental factor qualifiers: barriers and facilitators)[1]<br>F1 Mild facilitator<br>F2 Moderator facilitator<br>F3 substantial facilitator<br>F4 complete facilitator<br>Same coding approach and descriptors for barriers B1,B2, B3, B4	F1-F4<br>B1-B4<br>8 unable to determine (insufficient information)<br>9 not applicable
Attitude Family year 5 and 10		
Attitude Case manager year 5 and 10		
Case management interventions (actions)	Lukersmith et al (2015) Case Management Taxonomy (CMTaxonomy)[2-4] Interventions – 9 main actions (parent categories) and the holistic assessment and coordination action (child categories)	ACTIONS<br>• Assess 1 (measure outcomes, test and observe action categories)<br>• Assess 2 – gather information and listen)<br>• Planning – proactively and person centred planning to identify their goals<br>• Education<br>• Training and skill development<br>• Emotional and motivational support<br>• Advising<br>• Coordinate 1 – navigate, facilitate, advocate, collaborate<br>• Coordinate 2 – case consult, maintain feedback, manage documentation<br>• Monitoring<br>• Related actions (grandchildren categories)
Case management intensity	Lukersmith et al (2015) Case Management Taxonomy (CMTaxonomy)[2, 3] Service tree (inputs) intensity classification	1. Low intensity < 3 times per month per participant<br>2. Medium 1-3 times per week, per participant<br>3. High intensity (> 3 times per week with the participant)
Phase post injury	My Plan Phases post injury [5]	1. Continuing my recovery (adjusting to & settling into living at home, doing activities<br>2. My participation (participating in my chosen activities and major life areas - education, work, community, social life)<br>3. Maintaining my way of life (living life, maintaining my health and wellbeing)
Goal target concept	World Health Organization ICF (environmental factor qualifiers: barriers and facilitators)[1]	Goal concept mapped to the ICF using the ICF browser and the 2 level ICF code. (if not possible because poor goal description then one level code used).<br>The ICF Linking rules were applied [6]
Goal and step rating	Community Living Plan (CLP) or My Plan Goal rating [5]	1. Not achieved<br>2. Partially achieved<br>3. Achieved<br>4. W withdrawn or did not proceed

1. World Health Organization (WHO). *International classification of functioning, disability and health*. 2001; Available from: https://apps.who.int/iris/handle/10665/42407.
2. Lukersmith, S., et al., *The brain injury case management taxonomy (BICM-T); a classification of community-based case management interventions for a common language.* Disability and Health Journal, 2015. **9**(2): p. 272-80.
3. Lukersmith, S., *: A Taxonomy of Case ManagementDevelopment, Dissemination, and Impact.* , in *Faculty of Health Sciences*. 2017, University of Sydney: The Sydney eScholarship Repository, Post graduate theses/Sydney Digital Theses (Open Access).
4. WHO ICHI Development Project. *International Classification of Health Interventions (ICHI) Alpha 2 version* 2013; Available from: http://sydney.edu.au/health-sciences/ncch/about.shtml.
5. Lukersmith, S., et al., *My Plan – a new meso to micro level building block to promote person-centred care planning.* Int J Integr Care, 2017. **17**(5): p. A60.
6. Cieza, A., et al., *Refinements of the ICF Linking Rules to strengthen their potential for establishing comparability of health information.* Disability and Rehabilitation, 2016.
