# Supplementary figures and images for: A Realist Evaluation of Case Management Models for People with Complex Health Conditions Using Novel Methods and Tools—What Works, for Whom, and under What Circumstances?
Source: Int J Environ Res Public Health. 2023 Feb 28;20(5):4362. doi: 10.3390/ijerph20054362 (PMC10002263; doi:10.3390/ijerph20054362)

## Intervention tree (throughputs) of the community-based case management taxonomy

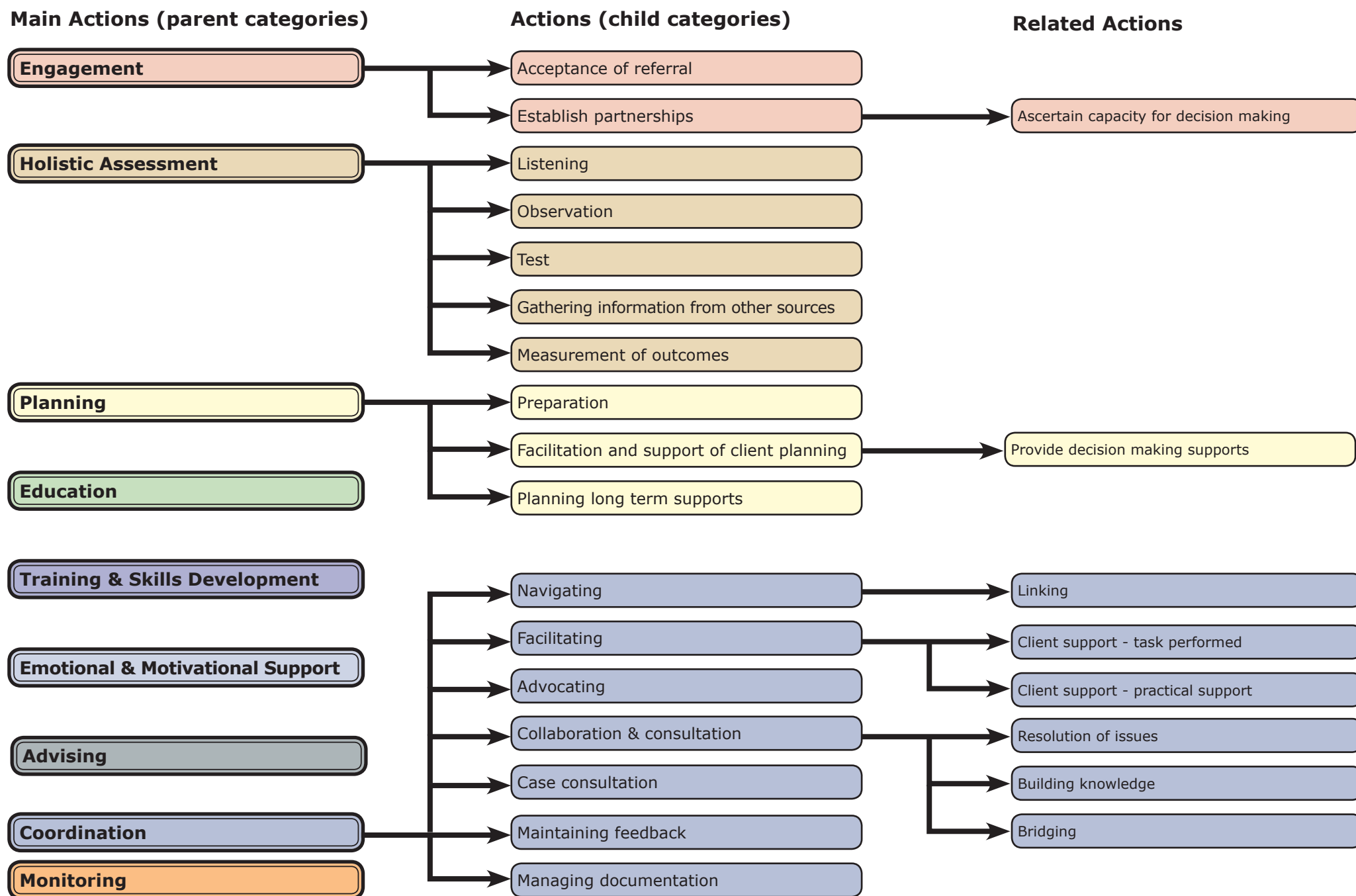

Supplement: Supplementary file 1 [file ijerph-20-04362-s001.zip › File S2 CMTaxonomy Intervention tree.pdf]

## Service tree (inputs) of the community-based case management taxonomy

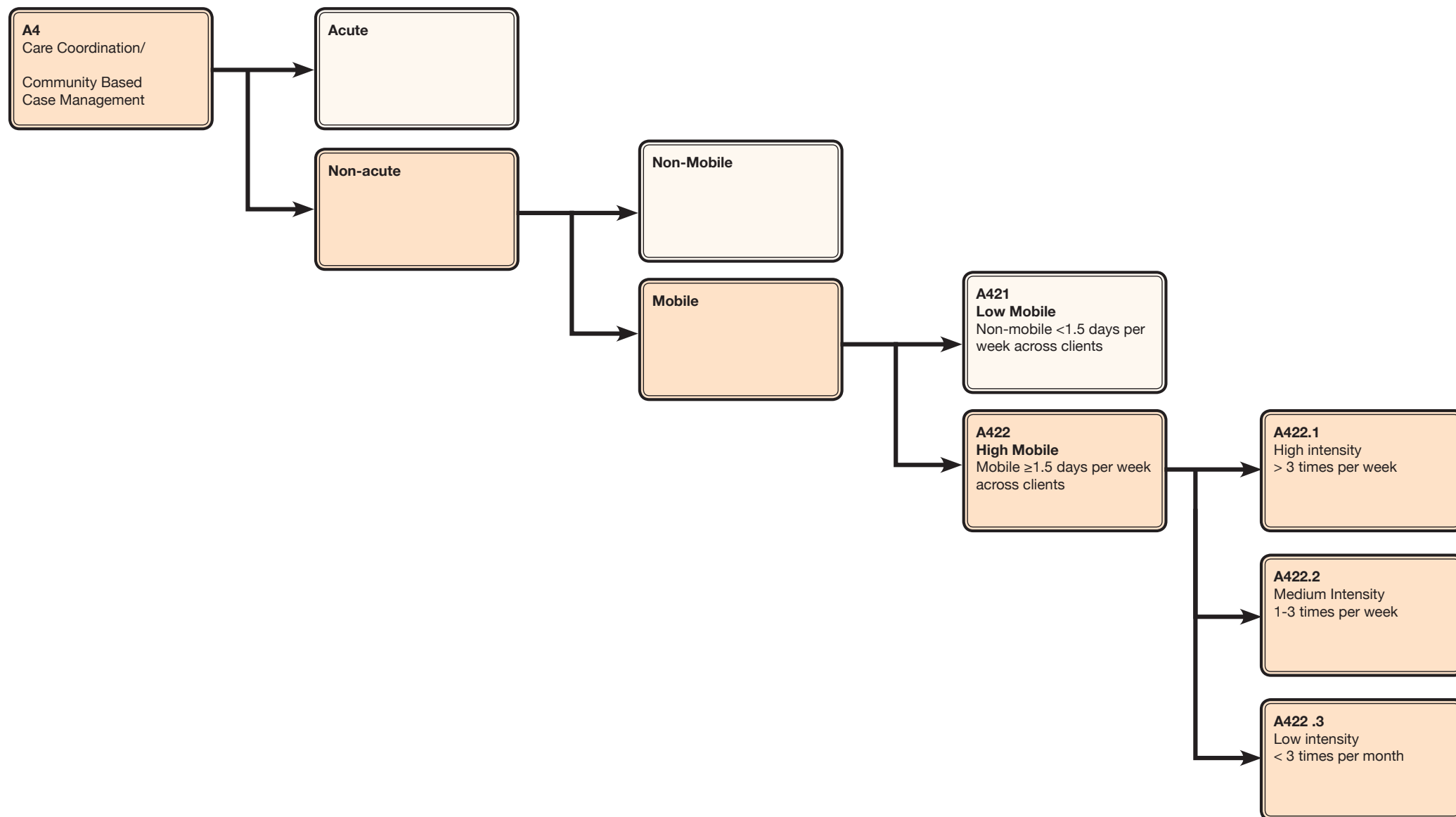

Supplement: Supplementary file 1 [file ijerph-20-04362-s001.zip › File S3 CMTaxonomy Service tree.pdf]
